# The British Journals: Anatomy and Physiology

**Published:** 1842-01

**Authors:** 


### II. THE BRITISH JOURNALS.

### ANATOMY AND PHYSIOLOGY.

Obliteration of the Aorta beyond the Arch. This is a long and not very interesting paper, the object of which is to point out two modes in which the aorta may suffer contraction or total occlusion. In the first case, the contraction or obliteration takes place a little below the point where the *ductus arteriosus* joins the aorta; and Dr. Cragie is inclined to think that this form of the affection is owing to the "obliterating action which has taken place in the *ductus arteriosus*, being from some peculiar cause prolonged into the aorta, and there giving rise first to contraction and then to obliteration of its coats." Subsequently he regards it as "the result rather of an arrest of development than of positive and active disease," though, in another part of the paper, he expresses himself as if the lesion were to be regarded as the result of inflammation. This form of contraction or obliteration of the aorta is distinguished from the one about to be noticed, by always occurring at the same point; by being due to some change in the coats of the vessel itself, not produced by external pressure as of a tumour, and by the smallness of the tract along which the lesion extends, which seldom or never exceeds a line.

The second form of constriction is stated to be owing to some "extrinsic" cause, as a tumour pressing on the vessel, or incurvation of the spine, or the pressure of an aneurism "formed on the arterial tunics:" and this form most usually takes place in the abdominal aorta, much further down than in the former case. From this last, it is further distinguished by the contraction being much more extensive than in the other, as it may occupy half an inch, an inch, or two inches.

In both these forms of aortal obliteration, the circulation in the lower half of the body is maintained through the lower intercostals, the epigastric and internal mammary arteries, and the circumflex arteries of the ileum.

The author seems to attach great weight to the distinction between the modes of obliteration described above; which distinction, however, so far as regards the occlusion simply, is obviously rather of pathological than of therapeutical interest: and the important conclusion, as it is termed by him, which the author deduces, viz. that the aorta, though at first sight essential to the circulation, may nevertheless be obliterated without fatal results is shown, by his own paper, to have been known since the year 1643.—Dr. Craigie, *in Edinburgh Med. and Surg. Journal, Oct.* 1, 1841.

Fœtus viable at six months. The catamenia of a lady flowed on the 24th of June, 1838, and continued for a week. She bore, on the 11th January following, a child which survived. The child was one of twins, the other twin died a few minutes after birth.—*Case by* A. Dodd, Esq., Surgeon to the Chichester Infirmary. *Provincial Medical and Surgical Journal.*

[The only ground of doubt in this case is, whether it was truly the catamenia which flowed in the end of June. It appears that the lady had had a great deal of "walking exercise" immediately before the appearance of that discharge, and it is conceivable that it may not have been truly menstrual. It is on the other hand proper to state that the lady herself was fully persuaded that it was the regular menses, and that her unusual intelligence and accuracy of observation rendered a mistake, on her part, unlikely; that any irregularity had never before happened to her; that her periods had always been entirely regular to an hour. Finally, the remarkably diminutive size of the child left no doubt as to its birth being singularly premature.]

On the Production of the Ergot of Rye. Mr. Quekett has already shown that ergot is owing to grain being infested with a parasitic fungus, in consequence of the sporidia of this fungus being by some means introduced into the interior of the plant, and ultimately arriving at the grain, which they find the most suitable matrix for their development.

Twelve healthy grains of rye, wheat, and barley, grown in contiguous fields in Surrey, were placed in a plate with a little water: some ergots of wheat were next introduced into the water; the sporidia of the fungus were detached by a camel-hair pencil, and the ergots then removed. After germination had made such progress, that the grains became wrinkled from appropriation of the albumen, the plants were removed into the country, and planted together in March last. A similar experiment was performed with the same number and variety of grains, but with fungus obtained from the exterior of an ergot of a large grass, *elymus sabulosus.*

The greater number of the grains treated as now described failed in being perfect plants. Only four of rye, (one infested with the fungus from the elymus, and three from that of the wheat), three of barley and four of wheat proved perfect. On every plant of rye, there are a greater or less number of ears possessing ergots: but in the barley there is only one imperfect ergot, and in the wheat there is none. Mr. Quekett remarks, that, if the cause of the ergot was in this instance of external origin, it is singular that the barley and wheat should have escaped; but explains the result on the supposition of rye being most liable to this peculiar infection.

Mr. Quekett conceives from these experiments that the production of ergot from the absorption of the sporules of the previously described fungus, by the fibres of the roots of the germinating grains, will be found to be the true cause of this singular production; and that, when they arrive at the grain, they convert it into the body known as the ergot.—Edwin Quekett, Esq., F.L.S., *Medical Gazette,* No. iii., *Oct.* 1841.

Motions of the Heart. A blue shark about eight feet long was caught, and brought on board the merchant ship Africa, in the Indian ocean, at 2, P.M. of the 15th July, 1836. After the seamen had cut open the body, and taken out the back bone, I removed the heart and held it in my hand. The contractions were slow, remarkably distinct, and in the following order:

1. The auricle.
2. The ventricle scarcely any interval.
3. A pause.

The contraction of the ventricle occupied a longer time (about double) than that of the auricle. The organ was empty. We had no instrument at hand to try if any sounds accompanied its motions, and the use of the finger employed by M. Cruveilhier did not occur to us.

These phenomena were continued for several minutes, and their order of succession was apparent, not only to Dr. Rumley, of the Ceylon Rifle Regiment, who was present, but to a number of military officers, all of whom, on being asked what part began the movements, uniformly pointed to the auricle.

The contractions were about fifteen in the minute at the beginning of the observation, and fell to about six when it was ended, the periods of repose being more lengthened in proportion to their relative duration than those of contraction.—Dr. Cavet, *Dublin Medical Press,* No. cxli., *Sept.* 15, 1841.

